# Cooperative breeding alters physiological and behavioral responses to habitat fragmentation

**DOI:** 10.1016/j.isci.2023.108717

**Published:** 2024-01-09

**Authors:** Beate Apfelbeck, Laurence Cousseau, Gladys Nyakeru Kung’u, Virginie Canoine, Janne Heiskanen, David K. Korir, Fredrick Lala, Petri Pellikka, Mwangi Githiru, Luc Lens

**Affiliations:** 1 Evolutionary Zoology Group, Department of Environment and Biodiversity, University of Salzburg, Hellbrunnerstr. 34, 5020 Salzburg, Austria; 2 Terrestrial Ecology Unit, Department of Biology, Ghent University, K. L. Ledeganckstraat 35, 9000 Ghent, Belgium; 3 Department of Behavioral and Cognitive Biology, University of Vienna, Djerassiplatz 1, 1030 Vienna, Austria; 4 Department of Geosciences and Geography, University of Helsinki, P.O. Box 64, 00014 Helsinki, Finland; 5 Finnish Meteorological Institute, P.O. Box 503, 00101 Helsinki, Finland; 6 Wildlife Works, P.O. Box 310, Voi 80300, Kenya; 7 Zoology Department, National Museums of Kenya, Museum Hill Road, Nairobi 00100, Kenya; 8 Wildlife Research and Training Institute, P.O. Box 842, Naivasha 20117, Kenya; 9 University of Nairobi, Wangari Maathai Institute for Environmental and Peace Studies, P.O. Box 29053, Kangemi 00625, Kenya; 10 State Key Laboratory for Information Engineering in Surveying, Mapping and Remote Sensing, Wuhan University, Wuhan 430079, China

**Keywords:** Ecology, Ornithology, Physiology, Evolutionary biology, Evolutionary ecology

## Abstract

Animals respond to habitat alteration with changes in their behavior and physiology. These changes determine individual performance and thus precede changes in population size. They are therefore hypothesized to provide important insights into how animals cope with environmental change. Here, we investigated physiological and behavioral responses of a cooperatively breeding bird, the placid greenbul (*Phyllastrephus placidus*), in a severely fragmented tropical biodiversity hotspot and combined these data with remotely sensed (LiDAR) environmental data. We found that individuals had increased glucocorticoid hormone levels when breeding in territories with low native canopy cover or located within small fragments. However, when breeding with the help of subordinates, breeders in low quality territories had similar glucocorticoid levels as those in higher quality territories. Our study shows that sociality may impact how well animals cope with environmental change and contributes to our understanding of the role of glucocorticoid physiology and behavior in response to anthropogenic change.

## Highlights

• Forest degradation increases corticosterone levels (CORT) in a forest specialist bird
• Increased CORT is associated with reduced canopy cover
• Cooperative breeders have lower CORT than pair-breeders in degraded territories
• Cooperative breeding may reduce physiological costs of breeding in degraded forests

## Graphical abstract

**Figure:**
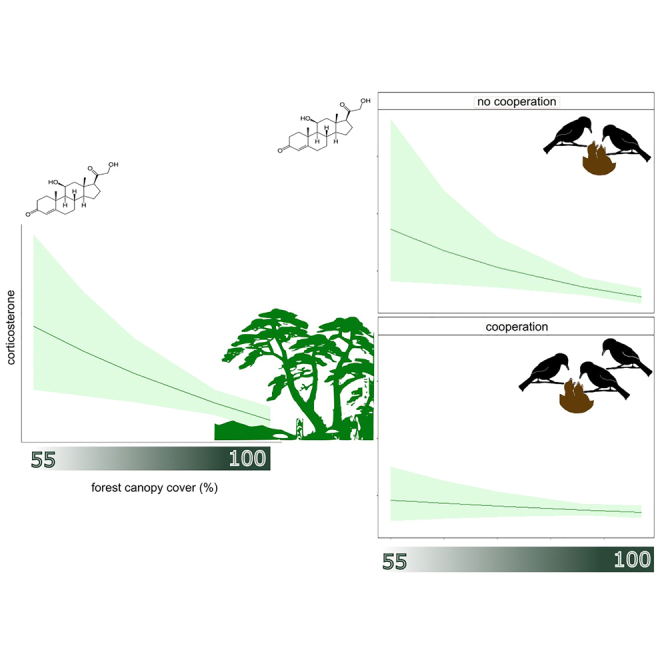

## Introduction

Endocrine control mechanisms represent key interfaces between the environment and the body condition of individuals and, therefore, may be used as proxies to indicate habitat quality from the perspective of the individual animal.^1^ These physiological systems may allow us to assess how well a species is coping with changes in environmental conditions, and to predict how such changes could affect trends in population abundance.^2^ Vertebrates respond to diverse challenges by activation of the hypothalamic-pituitary-adrenal (HPA) axis and the release of glucocorticoids.^3^ In birds, the glucocorticoid corticosterone coordinates the physiological and behavioral responses to both energetically demanding situations (baseline corticosterone) and potentially life-threatening events (stress-induced corticosterone) through binding to receptors of different affinity.^4^ Glucocorticoids are therefore one of the major mediators of the responses of animals to environmental change.^3^

Habitat fragmentation (i.e., the loss of continuous habitat) and habitat degradation change abiotic and biotic properties of the environment and, thereby, the availability of resources.^1^ For example, logging and other types of forest use affect forest size and structural forest properties, such as stand heterogeneity, canopy cover or the availability of dead wood, which can reduce the availability of food and nesting resources.^5^^,^^6^^,^^7^ Corticosterone levels have been found to be elevated in animals living in such disturbed forest sites,^8^^,^^9^^,^^10^^,^^11^ as well as in small-sized forest fragments.^12^^,^^13^ However, in some species no effect of habitat disturbance or quality has been found on corticosterone levels,^13^^,^^14^^,^^15^ indicating that responses may be species specific or context dependent, or may only appear when individuals are operating outside of their normal reaction scope, i.e., when the required energy exceeds the energy available in the environment.^3^ For example, species listed as threatened by the IUCN show stronger hormonal responses to forest degradation than least concern species^8^ suggesting that habitat specialists may be more affected by habitat degradation than habitat generalists.

Furthermore, elevations in corticosterone may only be found when energetic demands are high such as during parental care.^16^^,^^17^ In many vertebrates, baseline corticosterone concentrations are upregulated during breeding^18^ and can reflect parental workload during offspring provisioning.^16^ Elevated corticosterone levels or changes in other physiological markers particularly appear under experimental conditions, where workload is increased beyond the individual`s choice,^19^^,^^20^ or when food resources are limited.^21^ In fragmented and degraded landscapes, reduced prey biomass or increased travel distances^5^^,^^22^ could increase the daily energy expenditure of parents.^23^ Elevated baseline corticosterone levels may mediate the adjustments in metabolism and behavior that are necessary to meet the increased energy requirements when breeding in degraded habitat.^24^

While elevated baseline corticosterone levels can increase reproductive success through increased reproductive investment (“Cort-adaptation hypothesis”^25^), high stress-induced corticosterone levels can promote survival at the expense of reproduction during acute stressful events.^26^^,^^27^^,^^28^^,^^29^ When resources are limited and habitat quality is low, individuals may respond more strongly to acute stressors and favor self-maintenance over reproduction.^15^ Thus, the HPA axis is involved in differential investment into fecundity and reproduction and self-maintenance and survival, and these trade-offs may be especially apparent when resources are scarce.^24^

Many bird species live in social groups and it is likely, though not well studied, that both ecological and social factors shape habitat – hormone relationships.^30^^,^^31^ In cooperatively breeding species, where non-breeding subordinates assist to rear the offspring of the breeding pair, studies have shown that the dominant female can adjust her reproductive investment in eggs or nestlings to changing environmental and social conditions.^32^^,^^33^ This suggests that cooperative breeders possess high behavioral flexibility, which may enable them to cope with environmental change and variability^30^ without suffering reduced reproductive success.^34^^,^^35^ Thus, it can be expected that cooperative breeding may allow dominant individuals to successfully breed in degraded habitats without significant increases in parental workload and concomitant increases in corticosterone levels. Alternatively, increased resource competition in low quality habitat, may increase social conflict and thereby lead to increased corticosterone levels in group-living individuals, especially in subordinates.^36^^,^^37^

To test whether cooperative breeding can decrease the energetic costs of breeding in degraded habitats, we measured corticosterone levels of a cooperatively breeding forest specialist (placid greenbul, *P. placidus*) during four consecutive breeding seasons. Placid greenbul territories were located in native forest fragments that differed in size (1 ha–130 ha), native canopy cover, vegetation structure, and arthropod abundance.^38^

We tested the following predictions: (1) Higher foraging effort of individuals breeding in lower quality territories is reflected in higher baseline corticosterone levels; (2) Breeding females, which have the highest foraging effort,^39^^,^^40^ have higher baseline corticosterone levels than subordinates and breeding males; (3) Breeding pairs that receive help from subordinates (and therefore have lower foraging effort^32^) have lower baseline corticosterone levels than pairs without subordinates in low quality territories; (4) Subordinates have the highest baseline corticosterone levels if resource competition is more important^37^; (5) Individuals from lower quality territories have higher stress-induced corticosterone levels and take longer to resume feeding their nestlings (return latency) in response to capture and handling; (6) At nests with only one nestling parents mount a higher hormonal stress response and take longer to return than at nests with two or three nestlings, in particular in low quality territories. This was expected because hormonal and behavioral responses to stressors can be modulated by the value of the breeding attempt^41^^,^^42^ and one-egg clutches have reduced expected fitness gain, which has been shown to lead to infanticide in another cooperative breeder^43^; and (7) Individuals with higher corticosterone levels take longer to resume feeding after capture.

## Results

### Habitat fragmentation and degradation influences corticosterone levels

**Figure 1:**
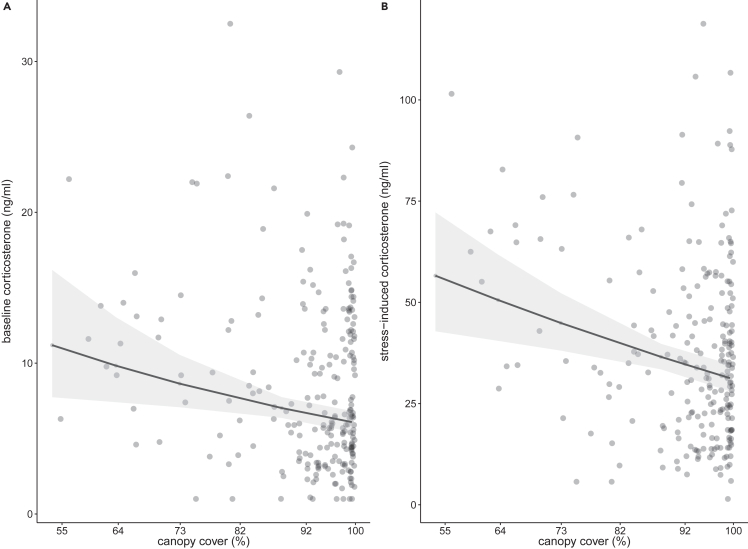
Corticosterone levels in relation to canopy cover (A and B) Placid greenbuls had higher baseline (A) and stress-induced (B) corticosterone levels (ng/ml) in territories with lower canopy cover (%). Shown are raw data points (dots), backtransformed REML model predictions (lines) and 95% confidence intervals (gray shading). Y axis scaling varies between (A and B). Model predictions for stress-induced corticosterone levels show estimates when baseline corticosterone was not included as covariate.

Territory quality was an important predictor of baseline and stress-induced corticosterone levels as native canopy cover (%) in the vicinity of the nest and fragment size were included within the topmost models during model selection, while the null model, which did not contain any measure of territory quality, was not. Models including vertical vegetation structure – another measure of territory quality - as well as models including the interaction between social status and territory quality were not within the top ranked models (baseline corticosterone) or the most parsimonious model did not include any interaction (stress-induced corticosterone). Models including canopy cover (%) received the strongest support. Baseline and stress-induced corticosterone levels were lower in territories with greater canopy cover or in larger fragments (Figure 1; Table 1). Subordinates of both sexes and breeding males had lower baseline and stress-induced corticosterone levels than females (Table 1). Sampling latency was positively related with baseline corticosterone levels, while maximum day temperature and time between setup of nets and capture (waiting time) showed a negative relationship with baseline corticosterone levels (Table 1). Inclusion of baseline corticosterone levels as a covariate in stress-induced models revealed that baseline corticosterone levels were strong predictors of stress-induced corticosterone levels (Table 1). While models including or excluding baseline corticosterone levels as a covariate showed qualitatively similar results, the relation between stress-induced corticosterone levels and canopy cover (%) was stronger when baseline corticosterone levels were not included.

**Table 1:** AICc model selection for baseline and stress-induced corticosterone levels with respect to fragment size, canopy cover and vegetation structure

Baseline corticosterone																			
Intercept	Nestling age	Sampling latency	Social status	Day temp	Wait time	Year		Fragment size	Veg struc	Canopy cover	Frag∗ social status	Veg∗ social status	Canopy∗ social status	df	logLik	AICc	Delta	Weight	Marginal R^2^/Conditional R^2^
																			Sample sizes (N)
2.06 [1.87; 2.25]	0.13 [0.02; 0.23]	0.20 [0.11; 0.29]	Male:−0.44 [−0.64;−0.24] Sub:−0.50 [−0.73;−0.27]	−0.13 [−0.23; −0.03]	−0.11 [−0.20; −0.02]	+				−0.13 [−0.22; −0.04]				14	−219.24	468.4	0.00	0.49	0.26/0.44 N = 231 N (nests) = 154 N (breeding groups) = 101
2.06 [1.87; 2.26]	0.10 [0.00; 0.21]	0.19 [0.10; 0.28]	Male:−0.41 [−0.61;−0.21] Sub:−0.48 [−0.71;−0.25]	−0.15 [−0.25; −0.05]	−0.12 [−0.21; −0.03]	+		−0.12 [−0.22; −0.03]						14	−219.66	469.3	0.83	0.33	0.26/0.43 N = 231 N (nests) = 154 N (breeding groups) = 101

**Stress-induced corticosterone**																			

Intercept	Nestling age	Baseline cort	Social status			Year	Number nestlings	Fragment size	Veg struc	Canopy cover	Frag∗ social status	Veg∗ social status	Canopy∗ social status	df	logLik	AICc	Delta	Weight	Marginal R^2^/Conditional R^2^ Sample sizes (N)
5.71 [5.20; 6.21]	−0.1 [−0.30; 0.11]	0.98 [0.78; 1.17]	Male:−0.76 [−1.18;−0.35] Sub:−0.56 [−1.04;−0.08]			+	0.18 [−0.26; 0.63]			−0.23 [−0.43; −0.03]				13	−365.89	759.5	0.00	0.33	0.48/0.60 N = 223 N (nests) = 149 N (breeding groups) = 99
5.7	−0.13	1.02	+			+	+	−0.05			+			15	−364.01	760.3	0.80	0.22	
5.7	−0.1	0.98	+			+	+			−0.12			+	15	−364.17	760.7	1.11	0.19	
5.7 [5.22; 6.23]	−0.14 [−0.34; 0.07]	1.01 [0.81; 1.2]	Male:−0.71 [−1.13;−0.29] Sub:−0.52 [−1.0;−0.04]			+	0.13 [−0.31; 0.56]	−0.19 [−0.39; 0.01]						13	−366.75	761.2	1.70	0.14	0.48/0.59 N = 223 N (nests) = 149 N (breeding groups) = 99

**Stress-induced corticosterone**																			

Intercept	Nestling age		Social status	Day temp		Year	Number nestlings	Fragment size	Veg struc	Canopy cover	Frag∗ social status	Veg∗ social status	Canopy∗ social status	df	logLik	AICc	Delta	Weight	Marginal R^2^/Conditional R^2^ Sample sizes (N)
5.67 [5.05; 6.30]	0.07 [−0.18; 0.32]		Male:−1.23 [−1.71; −0.75] Sub: −1.16 [−1.71; −0.61]	−0.16 [−0.40; 0.09]		+	0.39 [−0.14; 0.93]			−0.41 [−0.65; −0.17]				13	−406.44	840.6	0.00	0.74	0.27/0.48 N = 223 N (nests) = 149 N (breeding groups) = 99

In addition to the shown fixed factors, models also included nest identity and female identity as random intercepts. The variance accounted for by female identity was zero for baseline corticosterone levels, therefore, to calculate conditional R^2^, female identity was excluded. We report model selection for stress-induced corticosterone levels both when baseline corticosterone levels was included as covariate as well as when this covariate was not included in the models. Model selection was based on the dataset including all individuals (breeding males and females and subordinates). Only models within ΔAICc <2 are shown. Model estimates and 95% confidence intervals are shown for retained models.

Sub, subordinate, day temp, maximum daily temperature; frag, fragment size; veg struc, vegetation structure; canopy, canopy cover; Df, model degrees of freedom; AICc, Akaikés information criterion corrected for small samples sizes.

### Cooperation buffers the influence of habitat fragmentation and degradation on corticosterone

**Figure 2:**
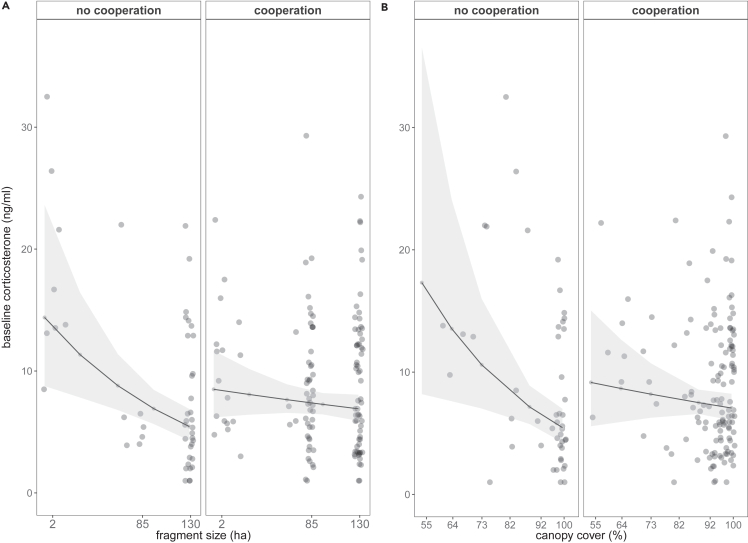
Cooperative breeding modulates glucocorticoid levels in response to habitat fragmentation and degradation (A and B) In small fragments (A) and in territories with low canopy cover (B), males and females breeding without subordinates had higher baseline corticosterone levels (ng/ml) than those breeding with the help of subordinates at the nest. Shown are raw data points (dots), backtransformed REML model predictions (lines) and 95% confidence intervals (gray shading).

A significant interaction between cooperation and fragment size or canopy cover indicated that females and males breeding without subordinates had particularly high baseline corticosterone levels in plots with low canopy cover and small sized forest fragments (Figure 2; Table 2). For stress-induced corticosterone levels, no significant interaction between cooperation or number of nestlings and fragment size or canopy cover was found (Table 2).

**Table 2:** Linear mixed model ANOVA results for the effect of cooperative breeding and territory quality on baseline (ng/mL, ln transformed) and stress-induced (ng/mL, sqrt transformed) corticosterone levels of breeding males and females

		Estimate	CI	df	F	p
**Baseline corticosterone**	**Intercept**	**2.04**	**1.82–2.25**	**150.1**		**<0.001**
	**Sampling latency**	**0.15**	**0.05–0.25**	**169.2**	**9.4**	**0.002**
N = 178	**Waiting time**	**−0.12**	**−0.22–**−**0.02**	**161.5**	**6.1**	**0.01**
N (nests) = 147	**Max day temp**	**−0.14**	**−0.25–−0.03**	**145.0**	**6.4**	**0.01**
N (breeding	**Sex (breeding male)**	**−0.36**	**−0.56–**−**0.16**	**97.9**	**13.0**	**<0.001**
groups) = 96	Cooperation	0.06	−0.18–0.29	128.7	0.2	0.6
R^2^ = 0.24/0.45	**Fragment size**	**−0.30**	**−0.47–**−**0.12**	**116.3**	**11.2**	**0.001**
	**Cooperation∗fragment size**	**0.23**	**0.02–0.45**	**114.0**	**4.7**	**0.03**
R^2^ = 0.22/0.49	**Canopy cover**	**−0.27**	**−0.44**−−**0.09**	**146.0**	**7.8**	**0.003**
	**Cooperation∗canopy cover**	**0.23**	**0.02–0.44**	**147.2**	**4.6**	**0.03**
**Stress-induced corticosterone**	**Intercept**	**5.75**	**5.00–6.51**	**110.7**		**<0.001**
	**Year 2017 2018**	**1.27**	**0.35–2.18**	**111.7**	**4.5**	**0.005**
N = 170	Year 2018 2019	0.11	−0.53–0.76			
N (nests) = 140	Year 2019 2020	0.95	0.25–1.64			
N (breeding	**Sex (breeding male)**	**−1.14**	**−1.66–**−**0.63**	**101.3**	**19.1**	**<0.001**
groups) = 93	Max day temp	−0.17	−0.46–0.11	121.2	1.4	0.2
R^2^ = 0.22/0.3	Number of nestlings	0.41	−0.17–0.99	95.1	1.9	0.2
	Cooperation	−0.08	−0.67–0.51	109.0	0.07	0.8
	**Fragment size**	**−0.31**	**−0.57**–**−0.06**	**99.7**	**5.8**	**0.02**
	*Nestlings∗fragment size*	*0.13*	*−0.43–0.68*	*92.2*	*0.2*	*0.7*
	*Cooperation∗fragment size*	*0.04*	*−0.53–0.61*	*78.7*	*0.02*	*0.9*
R^2^ = 0.25/0.32	**Canopy cover**	**−0.45**	**−0.70–**−**0.20**	**124.2**	**12.5**	**<0.001**
	*Nestlings∗canopy cover*	*0.12*	*−0.42–0.66*	*123.3*	*0.2*	*0.7*
	*Cooperation∗canopy cover*	*0.20*	*−0.35–0.75*	*122.9*	*0.5*	*0.5*

Models either included fragment size or canopy cover (%) and their interaction with cooperation. Values for explanatory variables are shown for the models with fragment size, but values for models including canopy cover were similar. For models with stress-induced corticosterone as the response variable, the interactions between fragment size or canopy cover and cooperative breeding or number of nestlings were not significant and therefore removed from the final models (shown in italics). Values in bold indicate p < 0.05. CI, 95% confidence interval; df, degrees of freedom; R^2^, marginal R^2^/conditional R^2^; N, sample size.

### The latency to return to the nest after capture depends on social status, fragment size, and brood value

The latency to return to the nest after capture and handling differed between breeding males and females, with the former returning significantly later than the latter (n = 176, events = 99, z = −3.41, p < 0.001). Breeding males and females were less likely to return within the video time in small fragments when only one nestling was present (number of nestlings ∗ fragment size: z = 2.22, p = 0.03), but the interaction between cooperation and fragment size was not significant (p > 0.05). The effect of baseline corticosterone levels on return latencies differed between breeding females and males (n = 118, events = 72, interaction log baseline corticosterone ∗ social status: z = 2.17, p = 0.03) in that females with higher baseline corticosterone levels tended to take longer to return to the nest, but males not. Stress-induced corticosterone levels did not predict the likelihood to return to the nest (n = 143, events = 83, z = −0.64, p = 0.52), neither in males nor in females (interaction square root stress-induced corticosterone ∗ social status: z = 0.77, p = 0.44).

## Discussion

Using data from four breeding seasons in combination with landscape and forest structure data, we provide first evidence that cooperative breeding can mitigate the energetic costs of breeding in low quality habitat. We show that individuals of a cooperatively breeding forest specialist had higher baseline corticosterone levels during nestling provisioning in smaller forest fragments and in territories with reduced canopy cover. However, in low quality territories, cooperative breeders had lower corticosterone levels than pairs breeding without subordinates. Likewise, breeders in low quality territories responded stronger to stressors, i.e., they had higher stress-induced corticosterone levels. In addition, when the value of the breeding attempt was low, individuals breeding in small fragments took longer to resume feeding of the brood than when two nestlings were present or in larger fragments. With respect to social status, breeding females had higher corticosterone levels than males or subordinates reflecting that they carry the highest reproductive burden within the group. These results are in accordance with an increased parental workload, which is reduced by the presence of subordinates, but not increased social conflict over resources, in low quality territories.

### Habitat degradation increases corticosterone levels

Elevated baseline corticosterone levels in territories with low canopy cover or in small fragments likely reflect suboptimal habitat conditions that increase the costs of breeding for insectivorous forest specialists such as the placid greenbul. In degraded forest, foraging effort may be either increased through reduced native canopy cover or an impoverished forest structure, which decreases the available surface for foraging and supports fewer arthropods.^38^^,^^44^ As in the present study an effect of native canopy cover, but not of vegetation structure, on corticosterone levels was found, it is plausible to assume that reduced native canopy cover increases traveling time between the nest and foraging patches through increased edge habitat or canopy gaps. In accordance with this interpretation, we found larger home ranges and longer travel distances between foraging patches in the degraded forest fragment Chawia than in Ngangao, which is characterized by a generally more intact canopy cover with few canopy gaps (Kung`u et al., unpublished data). Similarly, great tits (*Parus major*) breeding in urban parks with large gaps in canopy cover have higher energy expenditures than great tits breeding in closed forest,^23^ European starlings (*Sturnus vulgaris*) cover longer distances to foraging patches in fragmented landscapes^22^ and blue tits (*Cyanistes caeruleus*) have higher baseline corticosterone levels when breeding in low-density oak forests.^11^ Higher baseline corticosterone levels during colder weather are also in agreement with an increased foraging effort^45^ as arthropod abundances are lower on colder days.^38^ Furthermore, individuals with high baseline corticosterone levels also had high stress-induced corticosterone levels. In low-quality habitats with high baseline corticosterone levels, an individual’s energy demands may exceed available resources when dealing with a stressor, leading to elevated stress-induced corticosterone levels (“allostatic load and overload”^3^). Similarly, blue tits breeding in low quality forest mounted a faster corticosterone response than individuals breeding in high quality forest sites.^15^ Thus, in low quality territories the trade-off between reproduction and self-maintenance may be shifted toward self-maintenance in stressful conditions.

### Cooperation affects corticosterone levels in low quality habitat

In many cooperatively breeding birds, including placid greenbuls, breeders decrease their provisioning rate when helpers are present.^32^^,^^33^^,^^46^ Thus, in cooperative groups the load of providing food to nestlings is spread among several individuals. Thereby, foraging costs in degraded territories may be minimized and baseline corticosterone levels may remain low as breeders can reduce the number of trips to the nest, and hence daily traveling time, despite increased travel distances due to canopy gaps. In contrast, we did not find an effect of cooperation on stress-induced corticosterone levels. Thus, group members mainly seem to impact how strongly breeders respond to stressors through the reduction of workload in breeders and thus lower baseline corticosterone levels. This suggests that placid greenbuls may be able to reduce physiological costs of breeding in low quality territory, such as increased oxidative stress or reduced immune function,^47^ through cooperative breeding. As a long-lived tropical species, this may have important fitness benefits. We have previously shown that cooperatively breeding males and females have higher annual survival than pair breeders.^40^ Whether these fitness benefits are retained in low quality territories remains to be tested, but the present results suggest that through reduced physiological costs, as evidenced by low baseline corticosterone levels, breeders from cooperative groups may be able to avoid the survival costs of breeding in small fragments.^48^

### The trade-off between helping and self-maintenance

In placid greenbuls and other cooperative species, subordinates commonly contribute less to nestling provisioning than the breeding pair.^40^^,^^49^ Lower baseline corticosterone levels in subordinates than breeders indicate that they can keep the physiological costs of helping low.^47^ Although some subordinates do not provide any help^40^ and therefore may have low corticosterone levels, because subordinates were caught at the nest, it is likely that they were indeed helping to feed the offspring of the breeding pair. Furthermore, long return latencies after capture suggest that subordinates may only help if their own survival is not jeopardized,^50^ i.e., when confronted with stressors, subordinates may suspend helping behavior. However, it should be noted that subordinates often experienced longer handling times when both females and subordinates were caught during the same catching event, which also had an influence on return latencies. In addition, in contrast to the breeding pair, subordinates were molting at the time of capture, which may be an alternative explanation for low corticosterone levels as corticosterone can negatively affect feather growth.^51^ Furthermore, it has been suggested that when food availability is low, competition over resources and breeding roles may increase.^36^ However, in this case, subordinates would have been expected to have higher corticosterone levels than dominant breeders. Similarly, studies on Florida scrub jays (*Aphelocoma c. coerulescens*), found that male and female subordinates had similar or lower corticosterone levels than breeders^52^^,^^53^ and corticosterone levels of breeders were related to food predictability and availability.^54^ Thus, in placid greenbuls and Florida scrub jays, corticosterone levels likely reflect reproductive effort instead of social conflict. However, further studies during the pre-breeding season, where breeding roles are most likely redistributed, are necessary to confirm this hypothesis.

### The trade-off between current and future reproduction

When faced with disturbances during breeding, animals must decide whether to continue the current breeding attempt or abandon it in favor of their own survival and thus future reproductive opportunities.^27^^,^^42^ We found that individuals delayed the return to the nest longer after capture when they were breeding in small fragments and only one nestling remained. Placid greenbuls lay two eggs (seldom three), but brood size reduction can occur when one egg does not hatch or when one of the nestlings is predated (unpublished data). This may indicate a lower value of the brood to parents and in combination with low habitat quality, where they experience increased energetic demands (i.e., increased baseline corticosterone levels), they may favor self-maintenance over the current breeding attempt.^42^ This fits well with findings on another cooperatively breeding bird, the grey-capped social weaver (*Pseudonigrita arnaudi*), where breeding females remove the remaining egg after a partial nest predation event.^43^

### Behavioral response to stressors and corticosterone

It has been suggested that stress-induced corticosterone levels mediate the decision of individuals to redirect their focus from reproduction to survival^26^ and thus stress-induced corticosterone levels may be correlated with behavioral responses after challenging events.^27^^,^^29^^,^^55^ We found a tendency that baseline, but not stress-induced corticosterone levels predicted the latency of females to return to the nest. The absence of a relationship between stress-induced corticosterone levels and behavior might be due to variation in handling time between individuals, i.e., the time between capture and release. Although individuals were bled within 15–16 min after capture, release times varied when several individuals had been caught. Thus, corticosterone levels most likely continued to rise after the second blood sample. This is substantiated by stress-induced corticosterone levels of five individuals that were bled after 30 min, resulting in considerably higher stress-induced corticosterone levels than after 15 min and nest abandonment in all these cases. This may suggest that corticosterone is involved in mediating the behavioral response to stressors, but further data are needed.

### Conclusions and limitations of the study

Our study suggests that cooperative breeding may be especially advantageous in low-quality territories, as breeders with subordinates at the nest had lower corticosterone levels than those with no subordinates when breeding in territories with low canopy cover. It is less clear whether subordinates also benefit from group living in fragmented, degraded forests as offspring tend to stay with their parents for a shorter period (i.e., advance their postnatal dispersal) compared to offspring from continuous forests.^48^ Thus, our data provide the first evidence that the high behavioral flexibility of cooperative breeders may buffer against increased reproductive costs incurred in fragmented and degraded forests. However, reduced benefits for offspring to stay with their parents may potentially erode cooperation. In addition, our data confirm that corticosterone levels reflect energetically demanding periods in the life cycle of individuals such as providing nestlings under poor habitat conditions. Furthermore, when low habitat quality is combined with additional stressors, such as disturbances from humans, predators or inclement weather, increased corticosterone levels due to low habitat quality may tilt the trade-off between reproduction and self-maintenance toward the later. This may severely impair the reproductive success of individuals and thereby population persistence. As the results of our study are mainly based on correlational data, discussed causal relationships remain hypothetical. Furthermore, our study system comprises relatively few forest fragments, which are mostly relatively small. Thus, future studies in other regions and experimental approaches that directly manipulate habitat quality or individual workload would be needed to confirm the universality of our findings and the causal relationships between forest fragmentation and degradation, social factors, and physiology and behavior of forest specialists.

## STAR★Methods

### Key resources table

**Table:** 

REAGENT or RESOURCE	SOURCE	IDENTIFIER
**Biological samples**		

Bird blood samples	Wild placid greenbuls	N/A

**Deposited data**		

Raw data	This paper	Mendeley data: https://doi.org/10.17632/h5ryx3k8zr.1

**Experimental models: Organisms/strains**		

Placid greenbul (Phyllastrephus placidus), live birds	Taita Hills, SE Kenya, 03°25′S, 38°20′E	N/A

**Software and algorithms**		

R (version 4.3.1)	R Core Team	https://www.rproject.org/

### Resource availability

#### Lead contact

For additional information and resource inquiries, please direct your requests to the lead contact, Beate Apfelbeck (beateanna.apfelbeck@plus.ac.at).

#### Materials availability

Apart from generating data, this study did not produce any novel reagents or materials.

#### Data and code availability

• Data: Data have been deposited at Mendeley Data and are publicly available as of the date of publication. The DOI is listed in the key resources table.
• Code: The paper does not report original code.
• Any additional information required to reanalyze the data reported in this paper is available from the lead contact upon request.

### Experimental model and study participant details

In four breeding seasons, from 2016 to 2020, blood samples were acquired from wild placid greenbuls (*Phyllastrephus placidus*) breeding in the forest fragments of the Taita Hills in Kenya. We obtained 237 blood samples from 185 individuals (breeding females and males and subordinates of both sexes) at 156 nests belonging to 101 different breeding groups. Some individuals, mostly dominant females, were caught several times: 31 individuals were caught twice, 6 females thrice, 1 female four times and 1 female seven times. Most of these were recaught in different breeding seasons, except for eight females and one male who were caught twice within the same breeding season during different breeding attempts. For 11 individuals no stress-induced sample was obtained. As placid greenbuls are facultative cooperative breeders, whether a breeding pair bred with subordinates or not in some cases changed between breeding seasons. Animals were treated according to the ethical standards outlined and approved by the National Commission for Science, Technology, and Innovation of Kenya (NACOSTI/P/20/7374, NACOSTI/P/16/61881/14065, NACOSTI/P/18/61881/18658, NACOSTI/P/19/61881/27686, NACOSTI/P/20/3322, NACOSTI/P/21/8581), by the Kenyan Wildlife Service - Biodiversity Research and Monitoring (KWS/BRM/5001), the National Environment Management Authority (NEMA) and the Ghent University ethical committee (EC2022-073).

### Method details

#### Study system and species

We studied placid greenbuls (*Phyllastrephus placidus*) in the fragmented cloud forests of the Taita Hills (SE Kenya, 03°25′S, 38°20′E), which is the most northern part of the Eastern Arc Mountains biodiversity hotspot.^56^ The Taita Hills comprise three larger forest fragments of native forest (Chawia, Ngangao and Mbololo; 86, 120, 185 ha, respectively), one large, but heavily degraded mixed forest (Vuria, ∼100 ha) and 11 tiny patches (<8 ha) in a mountainous region of 250 km^2^ where the native forest cover has been significantly reduced between 1955 and 2004, mainly due to the clearing of forests for small subsistence agriculture.^57^ The Taita Hills native cloud forests experience degradation through subsistence use, such as logging of pole-sized trees, firewood collection, and grazing at varying levels in different forest patches, which has an impact on forest structure and arthropod abundance.^38^ The native forests are surrounded by croplands of agroforestry type, and exotic plantations of pine, cypress, and eucalyptus.^57^

Placid greenbuls are insectivorous forest specialists and as such especially prone to habitat fragmentation and degradation.^58^ They breed in pairs or in groups of up to seven individuals in the understory of tropical cloud forests.^59^ Flocks are composed of the breeding pair and a varying number of related and, less often, unrelated male and female subordinates. Related subordinates, i.e., offspring of the breeding pair, may help the breeding pair to feed nestlings while both related and unrelated subordinates defend the nest against predators.^40^^,^^60^ Breeding females have the highest reproductive burden as they deliver more food to nestlings than breeding males or subordinates.^39^^,^^40^ Breeding females and breeding males that receive help during offspring provisioning reduce their own feeding rates^32^^,^^40^ and survive better than breeding females without subordinates^40^ indicating that subordinates may reduce reproductive workload. In the present study, we did not distinguish between helping and non-helping subordinates as the determination of the former is dependent on videos taken after hormone samples were obtained (see below). As predation rates in our study population are high, restriction of study nests to those for which videos were available would have severely reduced sample sizes. However, as group size and the actual number of helping subordinates are correlated, the presence of subordinates is used as a proxy of helping.^32^

#### Field protocol

Between 2016 and 2022, placid greenbul nests were monitored in three of the large fragments (Ngangao, Chawia, Vuria) and five small fragments between October and March. The status of nests was checked every four to five days until nests were determined to be inactive, predated or successfully fledged. At each nest visit, the number of eggs or nestlings was recorded and any adult individual near the nest was identified. When nestlings were 5–12 days old (mean age 7.5 days), adult individuals were caught with one or two mist nets (length 3–6 m) that were erected close to the nest between 8 a.m. and 2 p.m. Between 2016 and 2020, immediately upon capture, a small blood sample was taken to determine baseline corticosterone levels. In most cases, samples were taken within 3 min after capture (mean 162 s, range 86–400 s,^61^). To determine stress-induced corticosterone levels, birds were kept in a cloth bag (routinely used capture-restraint protocol to measure the stress response in birds,^62^^,^^63^) and another blood sample (∼60 μL) was taken after 15 min. For the first five birds in 2016, stress-induced samples were taken after 30 min. This led to abandonment of nests and stress-induced samples of these birds were not included in analysis. Blood samples were taken through venipuncture from the wing vein, collected into heparinized capillaries and stored on ice until return to the field station. Blood samples were centrifuged the same day, the amount of plasma measured with a Hamilton syringe and stored in 500 μL pure ethanol.^64^ Blood cells were stored separately in ethanol. All birds were fitted with numbered aluminum rings, a unique combination of three color bands, weighed and measured (body mass, wing length, tarsus length). After the second blood sample, birds were released, and the nest was observed with a video camera (Sony FDR-AX53 handycam) for 3 h to record whether and when individuals resumed feeding of nestlings after the stressor had passed. Video cameras were mounted on a tripod 1,5–2 m from the nest, usually within vegetation, and covered with a black rain cover for camouflage and protection from precipitation. Return latencies after capture and handling were also determined during the breeding season 2021–2022, but no blood samples for corticosterone were taken in that year. Also, we were not always able to obtain all measurements for all individuals, i.e., in some cases blood samples were obtained, but no video recordings of return latencies and vice versa. To determine group size and composition, we used a combination of video recordings and nest observations. Nests were recorded for 5–6 h with a video camera between 7 a.m. and 3 p.m. when nestlings were 8–10 days old. Furthermore, during ringing events (adults or nestlings), the nest surroundings were scanned by an observer for group members. After nestling ringing, we further played back placid greenbul distress calls for a maximum duration of 5 min and observed the reaction of the group. In videos and during observations, group members were identified by their color rings.

#### Corticosterone assay

Plasma concentration of corticosterone was quantified following the instruction of a commercially available Enzyme-Immuno Assay (EIA) (ENZO Life Science Corticosterone EIA Kit; Cat.No.ADI-901-097). Beforehand samples were extracted as following (see also^64^): Samples were centrifuged for 10 min at 3900 rpm, and supernatant was then pipetted into new extraction tubes and dried down under a N_2_ stream at 37°C. Subsequently 4 mL Dichloromethane and 500 μL ddH2 were added, vortexed and placed in fridge overnight. The following day samples were placed on a shaker for 30 min, centrifuged for 10 min at 3900 rpm and then freeze-decanted twice to separate aqueous from organic phase. Both organic phases of each sample were collected into a new glass tube, dried down under a N_2_ stream at 37°C and resuspended into 750 μL assay buffer provided by the company. Samples were corrected for dilution. Distribution of samples across plates was balanced for breeding seasons and all samples of the same individual (baseline/stressed) were analyzed in the same plate. The sensitivity of the assay was 27 pg/mL. Chicken Plasma (CP; extracted and non-extracted) was used as Control to calculate Coefficient of Variation (CV). Intra Assay CV of non-extracted CP replicates was 8 ± 4.5% (mean ± SDev). Inter Assay CV of extracted CP was 16.2% and non-extracted CP was <11%. Mean % CV of duplicates was 2.10 ± 0.5%.

#### Territory quality

Placid greenbuls are insectivorous understory birds that glean arthropods from leaves and bark. Thus, habitat quality is determined by the availability of native understory vegetation. We, therefore, assessed vertical vegetation structure, canopy cover (%) and fragment size (ha) in placid greenbul territories using both data collected on the ground and remotely sensed airborne laser scanning data (ALS) (see below). Vertical vegetation structure is the presence of vegetation in different height layers of the forest and was assessed exclusively within the forest. Canopy cover is the percentage of native forest cover around nest sites, which is reduced when nests are close to the forest edge or when there are canopy gaps. Fragment size reflects a larger spatial scale than vertical vegetation structure and canopy cover and was based on native forest boundary maps of the Taita Hills created from airborne remote sensing images.^38^^,^^57^^,^^65^

#### Vertical vegetation structure

To determine vertical vegetation structure, we assessed vertical vegetation heterogeneity (VVH) and a canopy cover index in four subplots per territory that is, a center subplot, based on placid greenbul nest locations, and three additional subplots, 50 m away from the center subplot (50 m South, 50 m Northeast, 50 m Northwest) in April/May 2021. In each subplot, the presence or absence of vegetation (1/0) within a circle of 0.5 m radius in five height intervals (0–1 m, 1–5 m, 5–9 m, 9–15 m, >15) was recorded at five points within a 15 m radius leading to a total of 20 vegetation records per sampling plot. We computed VVH as an estimate of the diversity of vegetation layers by calculating the Shannon-Wiener diversity index across the five vegetation height intervals and an index of canopy cover by summing all presences of vegetation above 9 m for the 20 sampling points per plot.^66^ VVH and canopy cover index were highly correlated (r = 0,83). As canopy cover index showed the higher variation, it was retained for further analysis as an estimate of vertical vegetation structure of the forest around nest sites and in the following we will refer to canopy cover index as vertical vegetation structure.

#### LiDAR based canopy cover

We used airborne laser scanning data to determine native canopy cover (%) at each nest site. LiDAR data were obtained in January–February 2014 and February 2015 from an aircraft using a Leica ALS60 sensor. The mean flying altitude was approximately 1450 m above ground level and the mean return density of the collected data was 3.4 points per m^2.^^67^ Using LAStools software (rapidlasso GmbH) we classified LiDAR points to ground points and non-ground points and computed a digital terrain model (DTM) at 1 m resolution. Furthermore, point heights were normalized using DTM to derive heights from the ground level. To calculate canopy cover, we extracted normalized point clouds for circular areas of 0.79 ha (50 m radius) around nest sites. A 3 m height limit was applied to separate ground/understory and canopy returns,^67^ and canopy cover was defined as a ratio of the first returns from canopy and all first returns.^68^ All nest sites were located within native cloud forest and had dense vegetation cover below 3 m, except in cases of major canopy gaps or when nests were near the forest edge. Canopy cover was calculated using lidR package^69^ in R environment.^70^

#### Climate and weather variables

We obtained 4 km by 4 km gridded daily weather data (precipitation, maximum and minimum temperature) from the Kenya Meteorological Department (KMD). The KMD compiles these weather data by blending ground collected data from 10 weather stations within Taita-Taveta County and gridded data from meteorological satellites. Blending and interpolation is done using the Background-Assisted Station Interpolation for Improved Climate Surfaces (BASIICS) tool based on the simple and ordinary kriging concept. To obtain breeding season summaries, we averaged daily temperatures from October to March.

### Quantification and statistical analysis

Statistical analyses were done in R version 4.3.1^70^ using the packages lme4^71^ and MuMIn.^72^ Visualizations were created using ggplot2,^73^ sjPlot,^74^ and effects.^75^ Using separate models for baseline and stress-induced corticosterone levels, we tested whether territory quality and the social status of the caught individual (breeding female, breeding male, subordinate) and their interaction had an influence on corticosterone levels using linear mixed models (LMM). Next, we tested whether any effects of territory quality on corticosterone levels were modulated by cooperative breeding (0 = no cooperation, i.e., breeding pair only; 1 = cooperative group with subordinates). We ran different models to test for effects of social status and cooperation, as subordinates were not included in the latter analysis (because they were always part of cooperative groups). For stress-induced corticosterone levels, we also tested for a potential interaction between territory quality and the number of nestlings. Proxies for territory quality were fragment size (ha), native canopy cover (%) and vertical vegetation structure (see model selection below).

Previous studies have shown that the time between capture and blood sampling (sampling latency^61^), and ambient temperature^76^ can influence baseline corticosterone levels and thus were included in all models. Because the time lapse between set up of nets and capture (waiting time) varied between individuals, we also included it in the models. In addition, we included breeding season year (2016–2017, 2017–2018, 2018–2019, 2019–2020), nestling age (5–12 days), and the number of nestlings (one, two (including also rare cases of three nestlings, n = 4 nests)). Body mass can also influence corticosterone levels,^77^ but we did not add it to the models because of its overlap with social status, i.e., males are heavier than females. When modeling variation in stress-induced corticosterone levels, sampling latency and waiting time were not included. Because baseline and stress-induced corticosterone levels have been shown to covary,^78^ baseline corticosterone was included as a covariate. As stress-induced levels were determined largely by baseline corticosterone levels, we also ran the models without baseline corticosterone levels as a covariate. In the first case, we tested whether after controlling for variation caused by baseline corticosterone, territory quality explained additional variation in stress-induced levels, while in the latter analysis we tested whether stress-induced levels in general relate to territory quality. Initial inspection of data,^79^ revealed six samples with baseline corticosterone >38 ng/mL, which were considerably larger than the rest of the samples and therefore removed from the analysis. Final samples sizes can be found in Tables 1 and 2. Continuous predictor variables were standardized to a mean of zero and a standard deviation of one. Corticosterone levels were log transformed (baseline) or square root transformed (stress-induced) to improve normality and homoscedasticity of residuals. LMMs were fitted with restricted maximum likelihood with Satterthwaite approximation to calculate denominator degrees of freedom. For each analysis, we calculated the Type III Sum of Squares F-statistic to determine significance levels of effects. We used the function ‘anova’ in lmerTest-package,^80^ which is implemented through the lme4-package.^71^ Visual inspection of plots of fitted values against residuals did not reveal violation of normality and homoscedasticity assumptions.

To determine whether fragment size, native canopy cover around the nest site or vertical vegetation structure explained variation in baseline and stress-induced corticosterone levels we ranked linear mixed models based on the lowest Akaike’s information criterion corrected for small sample sizes (AICc).^81^ In addition to the basic model (containing all explanatory variables as explained above, but no proxy of territory quality), six further models were built either including fragment size, canopy cover or vegetation structure or their interaction with social status. Models were fitted using the larger dataset where we tested for an effect of social status. Models were ranked according to their AICc and only models within ΔAICc of <2,0 were retained. For model selection LMMs were fitted with package ‘lme4’,^71^ using Maximum Likelihood.

To test whether the latency to return to the nest after capture and handling differed with social status, cooperative breeding, territory quality, number of nestlings, or corticosterone levels we ran Mixed Effects Cox Proportional Hazards Regression models using the packages survival, survminer and coxme.^82^ To account for variation in handling time (i.e., time between capture and release) we computed stratified models. We restricted the dataset to breeding males and females as subordinates never returned to the nest within the time of the video recording. Furthermore, in the few cases where individuals had been caught twice within the same breeding season, only one breeding attempt was retained. We started with a full model including breeding season year, sex, cooperative breeding, number of nestlings, fragment size, wing length and the interactions between fragment size and cooperative breeding and fragment size and number of nestlings. To avoid issues of nonlinearity of covariates, we included fragment size as a factor with two levels (large, small) and did not run models for canopy cover and vegetation structure.

Subsequently we tested whether baseline or stress-induced corticosterone levels predicted return latencies in breeding females and males using Mixed Effects Cox Proportional Hazards Regression models with an interaction between corticosterone levels and social status. For baseline corticosterone levels, we restricted the dataset to samples taken within 200 s. Baseline corticosterone levels were log-transformed and stress-induced corticosterone levels square root transformed to improve linearity with the estimated hazard. We checked the assumption of proportional hazards using cox.zph. In addition, we inspected Martingale and Deviance residuals for linearity assumptions and outliers. In all models (LMMs and Cox), we included female identity and nest identity as random intercepts to account for non-independence of individuals caught at the same nest or repeatedly in different years. Significance was accepted at p ≤ 0.05. Non-significant interactions and covariates were removed from models to reduce model complexity.
